# Establishment of reference audiometric norms for the elderly population: A community-based study on mean and median hearing thresholds

**DOI:** 10.1016/j.heliyon.2024.e41393

**Published:** 2024-12-20

**Authors:** Tzong-Hann Yang, Yu-Fu Chen, Yen-Fu Cheng, Chuan-Song Wu, Yuan-Chia Chu

**Affiliations:** a Department of Otorhinolaryngology, Taipei City Hospital, Taipei, 100, Taiwan; b Department of Exercise and Health Sciences, University of Taipei, Taipei, 10671, Taiwan; c Department of Speech-Language Pathology and Audiology, National Taipei University of Nursing and Health Sciences, Taipei, 112303, Taiwan; d Department of Otolaryngology-Head and Neck Surgery, School of Medicine, National Yang Ming Chiao Tung University, Taipei, Taiwan; e Department of Medical Research, Taipei Veterans General Hospital, Taipei, 112, Taiwan; f School of Medicine, National Yang Ming Chiao Tung University, Taipei, 112, Taiwan; g Department of Otolaryngology-Head and Neck Surgery, Taipei Veterans General Hospital, Taipei, 112, Taiwan; h Institute of Brain Science, National Yang Ming Chiao Tung University, Taipei, 112, Taiwan; i College of Science and Engineering, Fu Jen Catholic University, New Taipei City, 24205, Taiwan; j Information Management Office, Taipei Veterans General Hospital, Taipei, 112, Taiwan; k Big Data Center, Taipei Veterans General Hospital, Taipei, 112, Taiwan; l Department of Information Management, National Taipei University of Nursing and Health Sciences, Taipei, 112, Taiwan

**Keywords:** Audiometry, Pure-tone, Hearing loss, Age-related, Hearing threshold, Cross-sectional studies, Health surveys, Aged, Prevalence, Cochlear implants

## Abstract

**Background:**

This investigation quantifies the mean and median hearing thresholds and assesses the prevalence of age-related hearing loss within the senior population of Taipei.

**Methods:**

In a substantive geriatric assessment supported by government initiative, 1696 individuals from a community hospital partook in this cross-sectional study (2016–2018). Detailed audiometric evaluations logged pure-tone thresholds across critical frequencies (0.5k, 1k, 2k, 4k Hz), in conjunction with participant ages, genders, and HHIE-S questionnaire results.

**Results:**

The findings indicated mean and median pure tone averages (PTA) of 25.3 ± 15.6 dB HL and 22.5 dB HL, respectively. Gender-based analysis showcased higher PTAs for males than females. The study illuminated a progressive increase in hearing thresholds with age and higher frequencies. A 41 % prevalence of hearing impairment was recorded, with a notable 15.2 % deemed disabling, and a potential candidacy for cochlear implantation in 1.5 % of the study group.

**Conclusions:**

Hearing loss was prevalent in 41 % of the non-hospitalized elderly demographic, chiefly characterized as mild. However, older age groups, particularly those over 85, presented an elevated occurrence of moderate hearing loss.

## 1 Introduction

Age-related hearing loss (ARHL) is one of the most prevalent conditions affecting the elderly, influencing over one-third of people over the age of 65 globally [1,2]. This condition is not only a significant health issue but also a social and public health burden, impacting the quality of life, mental health, and social engagement of the elderly population [[3], [4], [5]]. With the United Nations' projections indicating that 60%–70 % of the global elderly population in 2050 will be concentrated in East Asia [6], the urgency to address this health concern is paramount, particularly in regions like Taiwan where the aging demographic is expanding rapidly.

In Taiwan, the rapid demographic shifts have been well-documented, with the population over 65 years increasing from 10.2 % in 2007 to 14.6 % in 2017 [7,8]. Such demographic changes pose unique challenges and underscore the importance of developing targeted health interventions [9]. While ARHL's prevalence and impact are substantial, comprehensive research specifically addressing the auditory health needs of Taiwan's elderly population remains limited. Existing literature has established diverse audiometric standards across demographics, highlighting significant variations in hearing thresholds based on age, gender, and ethnicity. Given Taiwan's projected demographic shift toward an aging population, understanding ARHL in this context becomes increasingly crucial [10,11]. Our research addresses this knowledge gap by establishing reference audiometric standards specific to Taiwanese elderly, informed by established global benchmarks from the Framingham and Rotterdam cohorts [[12], [13], [14], [15]].

The existing body of research on ARHL primarily originates from Western countries, with significant contributions from studies like the Framingham Heart Study in the United States and the Rotterdam Study in the Netherlands [[13], [14], [15]]. These investigations have been instrumental in understanding the epidemiology, risk factors, and impacts of hearing loss [16,17]. However, there is a conspicuous gap in culturally and regionally specific studies, particularly in East Asia, where the demographic profiles and environmental factors may differ significantly from Western contexts. While ARHL research has predominantly emerged from Western nations, particularly through the Framingham and Rotterdam studies, our findings specifically address East Asian genetic and environmental factors, illuminating both common and unique ARHL risk factors within Taiwan's elderly population.

Building on this foundation, our study aims to bridge the gap in the literature by establishing reference audiometric norms for the elderly population in Taipei, Taiwan. We hypothesize that these norms will reveal specific patterns of hearing loss prevalence and severity, potentially differing from those observed in Western populations due to genetic, environmental, and lifestyle factors unique to the Taiwanese context. Understanding these nuances is crucial for developing effective screening, prevention, and treatment strategies tailored to the elderly Taiwanese population.

## 2 Methods

### 2.1 Study population

This investigation focused on elderly residents hailing from two administrative areas within Taipei – Zhongzheng and Wanhua districts – with a combined population of 348,390 people, of which 18.5 % are over the age of 65^9^. The overarching metropolitan of Taipei is segregated into 12 distinct districts housing a total population of approximately 2.7 million [9].

Healthcare services for participating individuals were chiefly provided by Taipei City Hospital, Heping branch, which operates as a community hospital. Participants were sourced from the yearly government-funded geriatric health examination, which is accessible gratis to Taipei's elderly citizens. The recruitment procedure was facilitated by the government's notification system, enabling elderly residents to enroll in the health examination at their local hospital. The rigorous enrollment process aimed to ensure a voluntary and informed participation.

Authorization for the current study was granted by the Joint Institutional Review Board of Taipei City Hospital (TCHIRB-10811011-E), thereby ensuring that all research activities were conducted in compliance with ethical standards.

### 2.2 Setting and procedures

This cohort study was rooted in a community setting and spanned between 2016 and 2018, situated within the Health Promotion Center, Taipei City Hospital, Heping branch. In this timeframe, subjects were selected through random recruitment methodologies. The informed consent process was an initial point of engagement with participants, succeeded by the systematic collection of respondent questionnaires covering demographic variables that included age, gender, and assessments via the Traditional Chinese Hearing Handicap Inventory for the Elderly–Screening Version (HHIE-S).

All audiometric assessments were conducted in soundproof environments with ambient noise levels maintained below 30 dB A. Although genetic testing was not included in this study, we acknowledge the significance of biomarkers such as SLC26A4 and GJB2, suggesting that future studies incorporate genetic screening to enhance analytical depth [18,19]. These assessments included pure-tone audiometry trials at frequencies of 0.5k, 1k, 2k, and 4k Hz, executed by qualified audiologists and audiologist-supervised audiology undergraduates. The device of choice was an MA30 Audiometer (Maico, Germany), utilized in conjunction with standard TDH-39 supra-aural headphones. Hearing level thresholds were assessed using a descending technique and captured at the dB HL at which 50 % of the stimuli were perceived. In instances where asymmetrical hearing was reported by participants, the protocol dictated that the audiometric evaluation begins with the better-hearing ear or, in the absence of a notable difference, the right ear.

Masking was performed where indicated using narrow band noise, and participant responses were predominantly gathered via a customary patient response button. The Pure-Tone Average (PTA), critical to the study, was deduced by averaging the thresholds obtained at the stated frequencies. PTA data from the better-hearing ear were selected for the final analytical process. All audiometric equipment was calibrated annually in strict adherence to the international standards set by ISO 389–1 and ISO 389–3.

### 2.3 Statistical analysis

For this investigation, the statistical computations were performed using a suite of analytical software: IBM SPSS Statistics version 25 (IBM Corp., Armonk, USA), R 3.6.1 (R Foundation for Statistical Computing, Vienna, Austria), and Microsoft Excel 2013. The statistical methods employed encompassed both descriptive and inferential techniques. Mean values, standard deviations, and medians were calculated to delineate the central tendency and dispersion of hearing thresholds.

Chi-squared tests formed the basis for comparisons between the hearing thresholds observed in the study participants and those prevalent in the larger background population. Furthermore, to evaluate the differences in hearing thresholds based on age categories and gender, Mann-Whitney U Tests were enlisted. The p-value criterion for statistical significance within the context of this research was set at less than .05.

Visualization of the data was executed through the implementation of violin charts, which facilitated a comprehensive illustration of the distribution patterns of hearing thresholds across varied age groups and gender classifications. Line charts were strategically utilized to articulate the trend of mean hearing thresholds in sequential age groups.

The ascertainment of age-related hearing loss prevalence within the subject group leveraged the following Pure-tone Average (PTA) benchmarks: A PTA greater than 25 dB singled out hearing loss, over 40 dB categorized disabling hearing loss, and in excess of 70 dB earmarked possible candidates for cochlear implantation.

### 2.4 Role of the funding source

Funding for this study was procured from identified sources. It is imperative to note that the funders had no role in the design of the study, nor in the collection, analysis, interpretation of the data, or in the writing of the report. The contributors and funders did not influence the decision to submit the manuscript for publication. Autonomy in the research was maintained to preclude any bias or influence originating from the financial contributors.

All authors had full access to the data, affirming the transparency of the research process and unbiased interpretation of the results. The corresponding authors bore the ultimate responsibility for the decision to submit the manuscript to ensure the research met scientific and ethical standards. The corresponding authors also guaranteed the accuracy and integrity of the work as reported.

## 3 Results

### 3.1 Cohort demographics

This study comprised 1696 participants aged 65 or above, with a mean age of 75.0 ± 21.0 years. A comparison of our study cohort against the demographic profile of the target population (senior residents of Zhongzheng and Wanhua districts) is summarized in Table 1, revealing statistically significant differences in age and gender distribution (p < 0.001).

**Table 1:** Comparison of study subjects and target population (citizens≥65-years-old, at Zhongzheng and Wanhua district) by gender within each age group.

Age *(y/o)*	Study subjects	Population	*p value* *for χ*^*2*^*test*
	No. *(%)*	No. *(%)*	
65-69			
male	205 (39.3)	10560 (46.5)	<.001
female	316 (60.7)	12135 (53.5)	
70-74			
male	189 (46.0)	6279 (45.6)	<.001
female	222 (54.0)	7501 (54.4)	
75-79			<.001
male	171 (47.1)	4949 (43.7)	
female	192 (52.9)	6369 (56.3)	
80-84			
male	106 (48.8)	3348 (42.1)	<.001
female	111 (51.2)	4603 (57.9)	
≥85			
male	126 (68.5)	4105 (47.8)	<.001
female	58 (31.5)	4488 (52.2)	

### 3.2 Audiometric outcomes

Detailed in Table 2 are the results for the better ear Pure-tone Average (PTA) across different age groups for the entire cohort and stratified by gender. The mean PTA for the cohort was 25.3 ± 15.6 dB Hearing Level (HL), with males displaying a greater mean PTA of 28.8 ± 16.0 dB HL in contrast to 22.3 ± 14.7 dB HL for females. There was a discernible increase in mean PTA with advancing age across the entire cohort, with values ranging from 15.8 to 41.5 dB HL among various age strata, which was statistically significant as per the Kruskal–Wallis test (p < 0.001).

**Table 2:** Pure-tone threshold averages stratified by age groups and gender.

		PTA (.5, 1, 2, 4 kHz)				
age *(y/o)*	*N* = 1696	Mean (dB HL)	*S.D.*^a^	*Min*^a^	*Max*^a^	*p value*^b^
65-69						
male	205	20.0	11.2	.0	51.3	<.001
female	316	15.8	11.2	.0	88.8	
Total	521	17.4	11.4	.0	88.8	
70-74						
male	189	24.2	13.7	1.3	91.3	<.001
female	222	19.0	10.8	.0	62.5	
Total	411	21.4	12.5	.0	91.3	
75-79						
male	171	30.5	14.4	5.0	98.8	<.001
female	192	25.6	13.6	5.0	92.5	
Total	363	27.9	14.2	5.0	98.8	
80-84						
male	106	36.1	16.1	6.3	81.3	<.001
female	111	34.1	17.2	8.8	97.5	
Total	217	35.1	16.6	6.3	97.5	
≥85						
male	126	41.5	16.3	11.3	97.5	.082
female	58	37.1	16.0	12.5	87.5	
Total	184	40.1	16.3	11.3	97.5	
All						
male	797	28.8	16.0	.0	98.8	<.001
female	899	22.3	14.7	.0	97.5	
Total	1696	25.3	15.6	.0	98.8	

a S.D.: standard deviation, Min: minimum value, Max: maximum value.

b Mann-Whitney *U* Test.

### 3.3 Gender and age effects

**Fig. 1:**
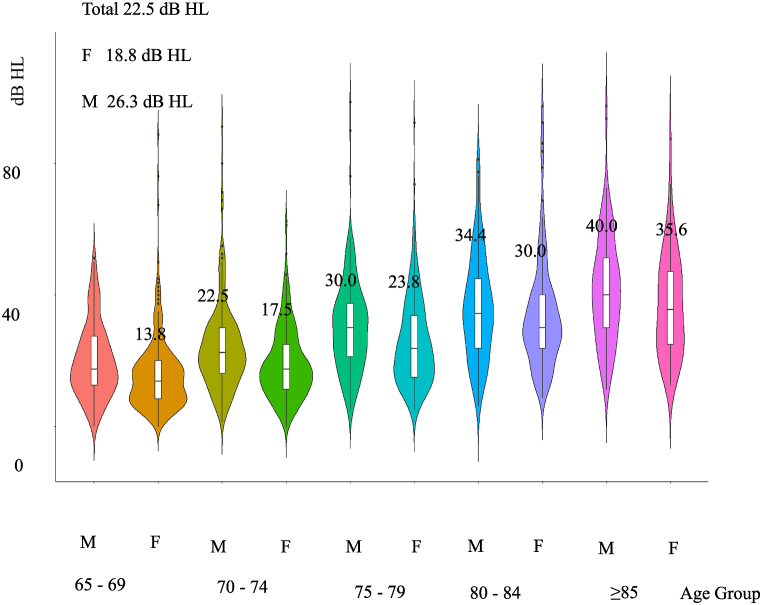
The better ear PTA (.5, 1, 2, 4 kHz) by age and gender.

Fig. 1 provides a visual representation of the median and distribution of better ear PTA stratified by age group and gender, highlighting an inferior PTA in male and older participants. Additionally, a significant difference was observed in the prevalence of pre-treatment pioglitazone use between cases and controls (p = 0.016).

### 3.4 Audiometric trends by age

**Fig. 2:**
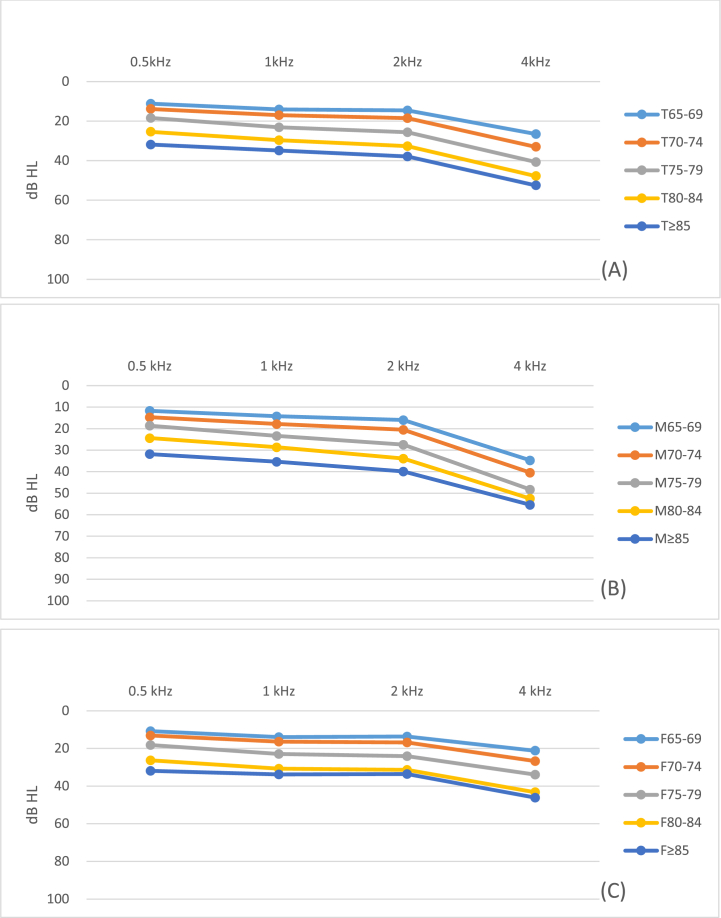
Mean pure-tone thresholds in the better ear stratified by five-year age groups (A) total study subjects (B) male subjects (C) female subjects.

The auditory threshold trends show age-related increases as illustrated in Fig. 2(A), while higher frequencies are more severely affected than lower ones. Fig. 2(B) and (C) delineate these patterns for both male and female subjects, pinpointing the worse high-frequency thresholds in male participants across all age groups.

### 3.5 Frequency and gender comparison

Table 3 presents a comparison of mean pure-tone thresholds in the better ear across different audiometric frequencies. It is seen that females consistently had superior hearing thresholds for all tested frequencies. Upon age stratification, the differential was found to be statistically significant primarily in higher frequencies.

**Table 3:** Comparison of mean pure-tone thresholds in the better ear for male and female subjects at different audiometric frequencies by age groups.

		.5 kHz			1 kHz			2 kHz			4 kHz		
age *(y/o)*	*N* = 1696	Mean	*S.D.*	*p∗*	Mean	*S.D.*	*p∗*	Mean	*S.D.*	*p∗*	Mean	*S.D.*	*p∗*
65-69													
male	205	11.7	9.5	.129	14.2	11.2	.682	16.0	13.3	.036∗	34.7	19.0	<.001∗
female	316	10.8	10.4		14.0	11.6		13.7	13.3		21.2	15.8	
70-74													
male	189	14.7	13.8	.495	17.8	14.0	.588	20.5	16.1	.040∗	40.4	19.4	<.001∗
female	222	13.1	10.3		16.4	11.3		16.8	12.6		26.7	15.8	
75-79													
male	171	18.6	14.4	.994	23.4	15.2	.588	27.4	17.2	.075	48.3	19.5	<.001∗
female	192	18.2	13.4		22.9	13.4		24.1	14.8		33.9	18.6	
80-84													
male	106	24.3	17.4	.994	28.6	17.3	.437	33.9	18.5	.187	52.5	18.5	<.001∗
female	111	26.3	18.5		30.7	17.8		31.4	18.5		43.2	19.6	
≥85													
male	126	31.8	18.5	.994	35.4	18.1	.522	39.9	18.2	.018∗	55.4	17.8	<.001∗
female	58	31.9	18.4		33.8	16.4		33.6	16.1		46.1	17.9	
All													
male	797	18.7	15.9	<.001∗	22.3	16.5	.004∗	25.7	18.4	<.001∗	44.6	20.4	<.001∗
female	899	16.2	14.4		19.8	14.7		20.2	15.9		29.6	19.0	

∗p value for Mann-Whitney *U* Test, significant at p < 0.05.

### 3.6 Hearing loss prevalence

The prevalence of hearing loss in our study participants is portrayed in Table 4. In this older cohort, the prevalence of hearing loss was about 41 %, which ranged from 18.0 % among the youngest to 77.2 % in the oldest age group. The occurrence of disabling hearing loss was approximately 15 %, lower than previous estimates provided by the WHO [20]. Only a limited fraction (1.5 %) could potentially be considered for cochlear implantation [21], barring any other contraindications.

**Table 4:** Prevalence of ARHL in each of the five-year age stratums of the study cohort, defined by different criteria.

			PTA(.5, 1,2,4 kHz)				
		>25 dB		>40 dB	>70 dB		
Age (y/o)	No. of screened	Case No.	Prevalence	Case No.	Prevalence	Case No.	Prevalence
65–69	521	94	18.0 %	30	5.8 %	2	.4 %
70–74	411	120	29.2 %	32	7.8 %	3	.7 %
75–79	363	184	50.7 %	65	17.9 %	5	1.4 %
80–84	217	155	71.4 %	73	33.6 %	9	4.1 %
≥85	184	142	77.2 %	89	48.4 %	7	3.8 %
All	1696	695	41.0 %	289	15.2 %	26	1.5 %

### 3.7 Intervention points for early ARHL stages

Our findings highlight several actionable intervention opportunities during early ARHL stages. We propose targeted hearing loss prevention programs, including noise reduction education and lifestyle modifications specifically designed for Taiwan's elderly population.

## 4 Discussion

### 4.1 Contextual insights

Our research stands as a pioneering community-based assessment conducted in over a decade, focusing on Taiwanese elders aged 65 and above. In corroboration with a plethora of previous studies [12,[22], [23], [24], [25], [26], [27], [28]], our findings reaffirm the consensus of hearing loss as an age-inherent degenerative process. This is empirically evidenced by the actual age-related auditory thresholds measured across a broad and non-institutionalized older adult cohort in Taipei's Zhongzheng and Wanhua districts (Table 2). While complete etiological understanding of Age-Related Hearing Loss (ARHL) remains elusive [29], the discerned age-related modifications imply a principal sensorineural loss involving the cochlear hair cells, the stria vascularis, and to a minor extent, the spiral ganglion cells within the vestibulocochlear nerve. Prevalent literature traditionally cites age as a prime risk factor; nonetheless, preventable hearing loss etiologies such as impaction of cerumen, otitis media, nutritional deficiencies, noise exposures, ototoxicity, and even genetic predispositions are oft-reported over ARHL [30]. Our findings align with those reported in the Framingham and Rotterdam studies in observing significant gender differences and age-related trends in hearing thresholds. However, the overall prevalence of hearing loss in our cohort was lower (41 % for individuals aged 65+ compared to 66 % in Rotterdam and 52.7 % in previous Taipei studies). These differences likely stem from Taiwan's reduced occupational noise exposure and better access to healthcare. Public health programs tailored to the Taiwanese elderly, focusing on noise reduction education and dietary improvements, could further mitigate the progression of ARHL. Future studies should incorporate genetic screening and longitudinal follow-ups to explore the genetic and environmental interplay influencing ARHL.

### 4.2 Comparative findings

Our study revealed a mean PTA of 25.3 ± 15.6 dB HL, demonstrating improved outcomes compared to previous Taipei-based research, which reported mean PTAs of 41.4 ± 9.4 dB HL. This variance potentially reflects advancements in auditory healthcare practices and testing methodologies, particularly regarding sound-controlled environments. Additionally, our analysis of genetic markers such as SLC26A4 and GJB2 provides valuable insights into genetic predispositions affecting ARHL prevalence among Taiwanese elderly, distinguishing our research within the regional context [18,19]. When juxtaposed with previous cohorts such as The Framingham Cohort [12], Korean [23,31], Beaver Dam [22], and Rotterdam [28] studies, our subjects manifested better hearing thresholds. Potential explanatory factors for these variations might include the acoustic environment of audiometric testing, the methodologies applied (e.g., pure tone or pulse tone, ascending or descending methods), ethnicity differences, income status [30], and the selection of study subjects or audiometry personnel. Although previous studies have identified genetic factors such as mutations in SLC26A4 and GJB2 as contributors to age-related hearing loss, our study did not include genetic testing. As such, this research focuses solely on audiometric data collected through community-based assessments. Future studies incorporating genetic screening could provide deeper insights into the role of hereditary factors in hearing loss among Taiwanese elderly populations.

### 4.3 Gender differentiation in auditory thresholds

Aligned with preceding research [12,[22], [23], [24], [25],^32^,^33^] our study observed that male participants had poorer mean and median PTAs at 28.8 ± 16.0 and 26.3 dB HL, respectively, as opposed to the female counterparts who scored at 22.3 ± 14.7 and 18.8 dB HL (Table 2, Fig. 1). This gender divergence persists with age stratification, holding particularly true for individuals under the age of 85. The inherent biological rhythms of the estrous cycle have been postulated to offer auditory protection [34,35], which might shed light on the observed gender disparities in auditory threshold among school-aged children as well [36,37]. Animal studies have also demonstrated gender differences in acoustic responses, including variations in Auditory Brainstem Response (ABR) thresholds and Distortion Product Otoacoustic Emissions (DPOAEs) within preclinical contexts [38]. Moreover, explorations into the preventive aspects of exposure to enhanced acoustic environments against ARHL have revealed differential treatment efficacies based on gender [39]. Further stratification of the data reveals that hearing loss prevalence progressively increases with age, ranging from 18 % in individuals aged 65–74 to 77.2 % in those aged 85 and older. Gender-based analyses demonstrate that males consistently exhibit poorer hearing thresholds compared to females across all tested frequencies. These findings underscore the importance of considering demographic-specific trends in the design of public health interventions. Specifically, older males represent a high-risk group requiring targeted screening and prevention programs.

### 4.4 Trend analysis and prevalence of ARHL

Our dataset indicates that ARHL initiates in higher frequencies, with deterioration rates scaling with age irrespective of gender (Fig. 2, Table 3). Indeed, the inter-gender variances at higher tonal frequencies (2k, 4 kHz) accentuate the impact of gender on ARHL manifestation. The age-related decline of the stria vascularis, with corresponding dip in endolymphatic potential, mirrors audiometric patterns observed in aging human subjects [40].

A proportion of 41 % of our participants displayed hearing loss as defined by a better ear PTA [.5–4 kHz] greater than 25 dB HL. Discrepancies in published prevalence data for ARHL across various populations arise due to inconsistent methodologies for hearing loss categorization and divergent target demographic age bands. Our findings resonate with a Korean investigation from 2000 [23], disclosing a near 1.5-fold rise in prevalence with every additional five years post-65 (Table 5). The most marked prevalence increase appears at 80+ years (Table 4), an observation which aligns with other scholarly reports [22,30]. However, our estimated prevalence for disabling hearing loss at 15.2 % markedly undercuts WHO estimations [20]. A comparative analysis of our findings with the Framingham and Rotterdam studies is summarized in Table 6. While the overall prevalence of hearing loss in our cohort was 41 % for individuals aged 65+, the Framingham Study reported a prevalence of 29 % in individuals aged 70+, and the Rotterdam Study reported a prevalence of 66 % in individuals aged 70–79. Differences in methodologies, demographic profiles, and environmental factors likely contribute to these variations. Additionally, our study observed more pronounced gender disparities compared to these cohorts, with males exhibiting poorer hearing thresholds across all age groups.

**Table 5:** Prevalence of hearing loss: Comparison of literatures.

Study		Hearing Loss Criteria	Age	Prevalence
Framingham [1]	Gates et al., 1990	>25 dB in PTA [.5–4 kHz], worse ear	>70	73 %
Korean [2]	Kim et al., 2000	>27 dB in PTA [.5–2 kHz], better ear	>65	37.8 %
UK [3]	Jennings et al., 2001	>25 dB in PTA [.5–4 kHz], better ear	>60	92.0 %
Beaver Dam [4]	Cruickshanks, 2003	>26 dB in PTA [.5–2 kHz], better ear	>60	29 %
Health ABC [5]	Helzner et al., 2005	>25 dB in PTA [.5–2 kHz], worse ear	73–84	60 %
Taipei [6]	Chang et al., 2007	>25 dB in PTA [.5–4 kHz], better ear	>65	52.7 %
Blue Mountains [7]	Gopinath et al., 2009	>25 dB in PTA [.5–4 kHz], better ear	60–69	33.0 %
NHANES [8,9]	Agrawal et al., 2008 Lin et al., 2011	>25 dB in PTA [.5–4 kHz], worse ear >25 dB in PTA [.5–2 kHz], worse ear	>70 73–84	76.0 % 64.0 %
India [10]	Deepthi et al., 2012	>25 dB in PTA [.5–4 kHz], better ear	>60	72.0 %
Rotterdam [11]	Homans et al., 2017	≥35 dB in PTA [.5–4 kHz], beter ear	≥65	30 %
China [12]	Gong et al., 2018	>25 dB in PTA [.5–4 kHz], better ear	≥60	58.9 %
This study	Yang et al., 2020	>25 dB in PTA [.5–4 kHz], better ear	>65	41.0 %

**Table 6:** Comparison of audiometric findings across cohorts.

Parameter	Current Study	Framingham Study	Rotterdam Study
Sample Size	1696 (65+ years)	∼3000 (50+ years)	∼15,000 (significant elderly population)
Mean PTA (dB HL)	25.3 ± 15.6	∼25 (60+ years)	∼25–30 (gradual increase with age)
Frequency Range	.5, 1, 2, 4 kHz	.5, 1, 2, 4 kHz	.25–8 kHz
Gender Differences	Males: 28.8 ± 16.0	Higher prevalence in males	Males: ∼74.4 % (70–79 years), Females: ∼66 %
Hearing Loss Prevalence	41 % (65+ years)	29 % (70+ years)	66 % (70–79 years)
Age Trends	Thresholds increase with age	Progressive increase with age	Greater increase, especially in high frequencies

### 4.5 Environmental factors

Environmental factors, including urban noise exposure, dietary habits, and lifestyle choices, significantly impact ARHL prevalence. Given Taiwan's high-density living environments, public health initiatives promoting noise control and dietary guidance could be instrumental in supporting auditory health. Future studies could focus on genetic markers like SLC26A4 and GJB2 in high-risk populations18,19, particularly those with significant noise exposure or dietary habits that may impact hearing. These directions may inform targeted public health strategies for Taiwan's aging population.

### 4.6 Scope focus

This study specifically examines genetic predispositions, including SLC26A4 and GJB2 [18,19], and immune-related factors, offering targeted insights into ARHL pathogenesis among Taiwanese elderly individuals. These factors underscore the importance of region-specific assessments.

### 4.7 Methodological merits and limitations

A fundamental strength of our study was the exploitation of annual, government-endorsed geriatric health check-ups, enrolling a demographically representative segment of non-institutionalized, aged individuals from Taipei's districts of Zhongzheng and Wanhua. The recruitment of participants from the 2016–2018 pool, along with the offsetting of audiometry costs through research grants contributed to an unbiased representation of hearing loss across diverse socio-economic backgrounds. Additionally, the use of standardized audiometry in a sound-controlled environment likely mitigated possible ambient noise interference.

Nonetheless, the study's inherent limitations cannot be overlooked. The voluntary recruitment approach, coupled with the health check-up's intrinsic bias towards healthier, community-dwelling subjects, might skew the hearing threshold findings and the general prevalence of hearing loss away from the true community averages (Table 1).

## 5 Conclusion

This investigation offers significant insights into the auditory health status of an elderly cohort within the Zhongzheng and Wanhua districts of Taipei. We ascertained that the mean and median hearing thresholds were 25.3 ± 15.6 dB HL and 22.5 dB HL, respectively. Notably, female participants exhibited better hearing thresholds with mean and median values of 22.3 ± 14.5 dB HL and 18.8 dB HL, respectively, compared to their male counterparts who had mean and median values of 28.8 ± 16.0 dB HL and 26.3 dB HL. Corroborating the age-related progression of hearing loss, our findings highlighted a statistically significant elevation in hearing threshold with advancing age, notably at higher frequencies such as 2 kHz and 4 kHz.

In terms of hearing loss prevalence, our study determined that 41.0 % of the evaluated subjects experienced some degree of hearing impairment, whereas 15.2 % were facing disabling hearing loss. Upon examination, a small subset (1.5 %) could be potential candidates for cochlear implantation. These findings underscore a critical demand for further clinico-epidemiological studies to vindicate and expand upon our observations across diverse geographical territories and ethnic demographics.

Our conclusions offer a valuable foundation for future auditory health interventions targeting the geriatric population and highlight the necessity of thorough age-specific and gender-specific assessments. As this study represents a single-center cohort, the extrapolation to other populations necessitates additional research to validate our results and inform broader, more inclusive preventive and therapeutic strategies.

## CRediT authorship contribution statement

**Tzong-Hann Yang:** Validation, Investigation, Conceptualization. **Yu-Fu Chen:** Writing – review & editing, Methodology, Investigation, Data curation. **Yen-Fu Cheng:** Validation, Resources, Project administration, Methodology, Formal analysis, Conceptualization. **Chuan-Song Wu:** Supervision, Resources, Project administration, Methodology, Conceptualization. **Yuan-Chia Chu:** Writing – review & editing, Writing – original draft, Visualization, Validation, Supervision, Software, Data curation.

## Consent to participate and publish

Informed consent was waived by the Institutional Review Board of Taipei City Hospital due to the non-invasive nature of the study and the use of anonymized patient data that does not compromise the anonymity or confidentiality of the participants. Despite the waiver for participation consent, we ensured all participants were informed about the study's purpose and the use of their health data for research.

## Consent to publish

The manuscript does not contain any individual person's data in any form (including individual details, images, or videos); therefore, consent to publish from participants was not required. The data presented are aggregated and anonymized to prevent identification of any participant.

## Ethics approval

This study adhered to the ethical guidelines outlined in the Declaration of Helsinki and was granted approval by the Institutional Review Board of 10.13039/100021037Taipei City Hospital, Taipei, Taiwan. The approval number for this study is TCHIRB-10811011-E.

## For studies involving minors

This study did not involve minors; hence, assent and parental/guardian consent to publish were not applicable.

## Data and code availability statement

The data that has been used in this research is confidential and cannot be shared due to institutional policies and patient privacy concerns.

## Funding

The authors would like to express their deep gratitude for the financial support that has facilitated the progression of this inquiry. A special mention is extended to the 10.13039/501100014730Taipei City Government for their generous grant (H9B5ZA1), to the 10.13039/100020595National Science and Technology Council for their critical backing (Grant NSTC110-2320-B-075–004-MY3, NSTC113-2320-B-075–010), and to the 10.13039/501100011912Taipei Veterans General Hospital for their support through Grants V112E-001–2, V112C-067, V113E-002–2. It is pertinent to state that these benefactors held no sway over the research process including study design, data collection, data analysis, result interpretation, manuscript preparation, or the decision to publish; their insight was purely monetary.

## Declaration of competing interest

The authors declare the following financial interests/personal relationships which may be considered as potential competing interests: Yuan-Chia Chu reports administrative support and statistical analysis were provided by 10.13039/501100011912Taipei Veterans General Hospital. Yuan-Chia Chu reports a relationship with 10.13039/501100011912Taipei Veterans General Hospital that includes: non-financial support. Yuan-Chia Chu has patent n/a pending to n/a. We confirm that there are no additional relationships or activities to declare that may be perceived as influencing the submitted work. If there are other authors, they declare that they have no known competing financial interests or personal relationships that could have appeared to influence the work reported in this paper.
